# Mapping the Landscape of Epigenetic Research in Diabetes Mellitus: A Decade-Long Bibliometric Analysis (2014-2024)

**DOI:** 10.2174/0115733998389913250807044815

**Published:** 2025-08-28

**Authors:** Yu’e Tang, Huan Zhu, Lele Liu, Xiuying Guo, Qianbo Zhang, Yuhe Da, Junqi Yang, Rifang Gu, Jidong Rong, Xuqiang Nie

**Affiliations:** 1 School of Clinical Medicine, Zunyi Medical University, Zunyi, 563006, China;; 2 College of Pharmacy, Zunyi Medical University, Zunyi, 563006, China;; 3 Key Lab of the Basic Pharmacology of the Ministry of Education & Joint International Research Laboratory of Ethnomedicine of the Ministry of Education, Zunyi Medical University, Zunyi, 563006, China;; 4 School Medical Office, Zunyi Medical University, Zunyi, 563006, China;; 5 Department of Cardiology, The Affiliated Hospital of Zunyi Medical University, Zunyi, 563006, China

**Keywords:** Molecular pathology, chronic hyperglycemia, DNA methylation, histone modification, research mapping, collaboration networks

## Abstract

**Introduction:**

Epigenetic regulation constitutes critical molecular mechanisms underlying the pathogenesis of diabetes and disease progression. While substantial mechanistic investigations exist, the field lacks systematic mapping of research trends, collaborative networks, and emerging frontiers.

**Objectives:**

To conduct the first comprehensive bibliometric evaluation of epigenetic studies in diabetes mellitus and its complications (2014-2024), identifying key research domains, international collaboration patterns, and innovative investigative directions to inform strategic research planning and highlight opportunities for innovative therapeutic approaches.

**Methods:**

We interrogated the Web of Science Core Collection using stringent inclusion criteria, analyzing 1,451 publications through advanced multi-dimensional metrics in CiteSpace (6.2.R4), VOSviewer (1.6.20), and Bibliometrix (4.1.3).

**Results and Discussion:**

A total of 1,451 original and review articles were retrieved, involving 83 countries/regions, 576 journals, and 7,645 authors. The United States produced the highest number of publications (n = 464), followed by China (n = 283) and Italy (n = 121). The *International Journal of Molecular Sciences* was the leading journal (66 publications), dominated by review articles (n = 53). Author collaboration networks were extensive, with Charlotte Ling emerging as the most prolific and influential author in publications, citations, and H-index. Keyword co-occurrence analyses emphasized type 2 DM, gestational DM, and diabetic nephropathy as primary research focuses, while new frontiers highlighted potential links to Alzheimer’s disease and fibroblast biology.

**Conclusion:**

This multi-dimensional analysis provides quantitative visualization of research evolution, delineates current investigative priorities, and highlights underexplored therapeutic targets. Our findings establish a strategic framework for transdisciplinary collaboration in precision diabetology.

## 1 INTRODUCTION

Diabetes Mellitus (DM) is a common chronic metabolic disorder marked by persistent hyperglycemia and is among the leading global causes of morbidity and death [1]. Recent projections by the International Diabetes Federation (IDF) estimate that 783.2 million individuals will be affected by 2045, underscoring its escalating prevalence [2]. This growing burden imposes significant strain on healthcare systems worldwide, driven mainly by both macrovascular complications (*e.g.*, coronary heart disease, stroke, peripheral vascular disease) and microvascular sequelae (*e.g.*, end-stage renal disease, retinopathy, neuropathy, and lower-extremity amputations) [3, 4]. Consequently, ongoing evaluations of research progress are essential to guide preventive strategies and therapeutic innovations.

Mounting evidence suggests that epigenetic alterations profoundly influence DM pathogenesis and progression. First conceptualized by Waddington in 1942, epigenetics encompasses heritable phenotypic changes that do not involve modifications in the DNA sequence itself [5]. Mechanistically, these changes span DNA methylation (particularly CpG island modifications mediated by DNMT3A/B), histone acetylation dynamics (HDAC/SIRT-regulated), chromatin remodeling, and regulation by non-coding RNAs, collectively modulating gene expression across developmental, stress-related, and pathological processes [6-9]. Emerging preclinical evidence demonstrates that transient epigenetic reprogramming can mitigate diabetic nephropathy progression [10, 11] and prevent hyperglycemia-induced endothelial memory [12]. Such discoveries position epigenetic modulation as a transformative therapeutic frontier in precision diabetology [13]. However, the exponential growth of research outputs necessitates systematic evaluation to identify high-value investigation domains.

Contemporary science mapping techniques address this need through computational topology analysis [14]. Unlike traditional reviews constrained by cognitive bias [15], bibliometric approaches enable: the quantitative assessment of international collaboration networks, temporal pattern recognition *via* keyword burst detection, and objective identification of conceptual clusters and emerging trends [16].

While epigenetic mechanisms contribute significantly to diabetic complications, they represent only one facet of a complex pathophysiological landscape that includes altered signaling pathways, inflammatory processes, oxidative stress, changes in osmolarity, and metabolic dysregulation—all primarily driven by hyperglycemia. Our bibliometric approach aims to map research trends within the epigenetic domain while acknowledging its position within this broader context.

Our study implements advanced bibliometric algorithms (VOSviewer, CiteSpace) [17, 18] to analyze 1,451 WoSCC-indexed publications (2014-2024).

By leveraging multi-level network visualization and temporal stratification techniques, we are able to: first, comprehensively map out the dynamic and evolving knowledge architecture within the realm of diabetes epigenetics. This mapping process provides a detailed overview of how knowledge in this field has been developing over time.

Secondly, we pinpoint underdeveloped research corridors that hold significant therapeutic potential. These are areas within diabetes epigenetics research that, despite their potential to yield important therapeutic breakthroughs, have not yet been fully explored.

Finally, based on our findings, we propose strategic priorities for next-generation epigenetic interventions. These priorities are designed to guide future research efforts and help in the development of more effective epigenetic-based treatments for diabetes.

Bibliometric analysis of epigenetic research in diabetes mellitus offers substantial practical value beyond traditional narrative reviews, serving multiple stakeholders in the scientific and healthcare communities. For researchers, our mapping of intellectual structures identifies critical knowledge gaps—particularly in non-methylation epigenetic mechanisms and tissue-specific modifications—while highlighting promising research directions at the intersection of emerging clusters. This strategic intelligence can guide investigative priorities and collaborative opportunities, potentially accelerating scientific discovery. For funding agencies, our analysis highlights areas of high impact and innovative potential, enabling evidence-based resource allocation decisions that maximize return on research investment. Notably, our identification of rapidly evolving research fronts versus established domains provides crucial context for strategic funding initiatives. For clinicians, this analysis reveals translational research priorities with immediate therapeutic implications, bridging the gap between basic epigenetic mechanisms and clinical applications by identifying epigenetic biomarkers and intervention targets approaching clinical utility. Finally, for journal editors and publishers, our work provides insight into the evolving research frontiers, publication trends, and influential works, informing editorial priorities and special issue planning. This comprehensive mapping of the intellectual landscape thus serves as a strategic compass guiding multiple stakeholders toward more efficient, impactful, and clinically relevant epigenetic research in diabetes.

In conclusion, this study delineates a clear trajectory for future research in the field of epigenetics as it pertains to diabetes. It aids researchers in identifying current hotspots and frontier areas, selecting appropriate collaborators, and provides guidance for journal selection to ensure high-quality submissions. Furthermore, it underscores the feasibility and potential of epigenetic therapies for diabetes, thereby paving the way for enhanced diagnostic and treatment options. This comprehensive mapping of epigenetic research in diabetes (2014-2024) offers critical insights that are unattainable through traditional narrative reviews. By identifying research hotspots, collaboration patterns, and emerging trends, our analysis establishes a strategic framework to inform future research priorities, funding allocations, and transdisciplinary collaborations in the domain of diabetes epigenetics.

## 2 MATERIALS AND METHODS

### 2.1 Study Design Overview

This work adopted a retrospective, descriptive bibliometric design aimed at mapping and quantifying epigenetic research in diabetes mellitus and its complications published between 1 January 2014 and 1 October 2024. The study followed the logic of the PRISMA-ScR workflow for literature identification, screening, and inclusion, while the analytic stages adhered to best-practice recommendations for each software package employed (CiteSpace 6.2.R4, VOSviewer 1.6.20, and Bibliometrix 4.2.1). The Web of Science Core Collection (all indices) served as the sole data source because of its broad coverage and stable citation-export functions. A topic search limited to titles, abstracts, and author keywords combined the terms “epigenetic*” or “epigenesis, genetic” with “diabetes” or “diabetic,” restricted to English-language articles and reviews published within the defined timeframe. This query returned 1,539 records. Following the automatic removal of four duplicates, two reviewers independently screened titles and abstracts to exclude editorials, meeting abstracts, proceedings papers, book chapters, notes, and items lacking author information; disagreements were resolved by consensus. The final analytical corpus comprised 1,451 publications—757 original research articles and 694 reviews.

### 2.2 Data Preparation and Standardization

Before analysis, all bibliographic fields were exported in plain-text and tab-delimited formats, then harmonized in Microsoft Excel. Journal titles and country names were standardized, author names were disambiguated using a VOSviewer thesaurus file, diacritics were removed, and a 10% random sample was manually cross-checked against the online Web of Science interface to ensure data integrity. Annual publication counts, total citations, and year-specific H-indices were plotted in GraphPad Prism 9.0. Co-authorship networks for countries, institutions, and authors (fractional counting, minimum of five items), as well as keyword co-occurrence maps (binary counting, minimum of ten occurrences; clustering resolution = 0.75), were generated in VOSviewer. CiteSpace produced reference co-citation networks, keyword timeline views, and burst-detection outputs using one-year time slices from 2014 to 2024, a g-index (k = 25), and Pathfinder pruning. Bibliometrix (*via* the Biblioshiny interface in R 4.3) delivered complementary analyses, including scientific production profiles, an author-publication timeline, and a thematic evolution map based on KeyWords Plus. All visual outputs were refined in Adobe Illustrator to meet journal layout requirements.

### 2.3 Analysis and Reproducibility

The study reports quantitative growth indicators (annual publications and cumulative growth rate), impact metrics (total citations, average citations per item, and H-index by year), and collaboration indices (link strength and proportion of multiple-country publications). Knowledge-structure parameters, such as modularity Q, silhouette scores, and keyword centrality, complement the depiction of the field’s intellectual architecture, while the strength and duration of citation bursts highlight emerging frontiers. Full search strings, raw data files, R scripts, and VOSviewer parameter files are provided in Supplementary File 1 and have been deposited in an open repository, whose DOI is listed in the Data-Availability Statement, ensuring complete reproducibility. Because the project analyzed publicly accessible bibliographic metadata without involving human participants or confidential information, institutional review board approval and informed consent were not required. Fig. (**1**) illustrates the process of literature search and data analysis.

## 3 RESULTS

### 3.1 Analysis Based on the Year of Publication

From January 1, 2014, to October 1, 2024, we identified 1,535 studies on epigenetics in diabetes and its complications in the WoSCC. After screening, 1,451 records remained: 757 original research articles and 694 review articles. As shown in Fig. (**2**), the annual publication counts have steadily risen over the past decade, reflecting continual scholarly interest. The first pertinent article was published in 2014 (n = 81), marking the decade’s lowest output. Subsequently, publication totals rarely fell below 100 per year, peaking at 169 in 2022. Although a slight dip (n = 134) occurred in 2023, overall activity remained robust. Between 2014 and 2019, citation frequencies and the H-index closely tracked publication trends. However, both metrics began to decline in 2020, with 2018 witnessing the highest citation and H-index values and 2024 recording the lowest.

### 3.2 Analysis of Countries

A total of 83 countries/regions contributed to this field (Fig. **3A**). The United States led with 464 publications (31.98%), followed by China (283, 19.50%), and Italy (121, 8.34%). Other key contributors included England (100, 6.89%), India (81, 5.58%), Germany (78, 5.37%), Sweden (77, 5.30%), Spain (70, 4.82%), Australia (69, 4.76%), and Canada (69, 4.76%). All countries in the top 10 each generated more than 50 publications (Fig. **3B**). Collaboration networks (Fig. **4**) demonstrated frequent partnerships, notably between China and the United States, the United States and the Netherlands, the Netherlands and Germany, as well as Sweden and Denmark. Table **1** displays citation frequencies and H-indices for the top 10 countries, with the United States (H = 69), China (H = 44), and Sweden (H = 38) ranking highest in overall impact.

### 3.3 Analysis of Journals

Our dataset covered 576 journals that publish on epigenetics in diabetes and its complications. Table **2** presents the top 10 journals by publication volume, which offers guidance for prospective authors. *The International Journal of Molecular Sciences* led with 66 articles, most of which were reviews (53). Notably, three of the top 10 journals maintained impact factors above 5, while eight exceeded 3. *Diabetologia* stood out with an IF of 8.4 and an SJR index of 3.355, highlighting its influence in the field.

### 3.4 Analysis of Authors

Overall, 7,645 authors contributed to research on epigenetics in diabetes and its complications. (Fig. **5**) shows the publication timelines of the 20 most productive authors, with Charlotte Ling and Tina Ronn experiencing the highest citation peaks in 2014 and 2019, respectively. Table **3** documents the 10 authors with the most publications, led by Charlotte Ling (30 total), who also commanded peak citation metrics and H-index values, demonstrating her central role in this domain. Collaboration patterns (Fig. **6**) varied, with several researchers collaborating primarily within their institutions. However, Alexander Perfilyev showed notable external collaborations across multiple centers.

### 3.5 Co-citation Analysis

Co-citation mappings (Fig. **7A**) revealed seven distinct clusters, the largest of which focused on diabetic nephropathy. Other thematic clusters included insulin secretion, diabetic retinopathy, type 2 diabetes, gestational diabetes mellitus, gestational diabetes, and life course development. (Fig. **7B)** highlights the top 20 references with significant citation bursts. Charlotte Ling’s 2019 article exhibited the strongest burst intensity (23.59), extending through 2024. Table **4** lists the 10 most frequently co-cited references, each surpassing 20 co-citations. Three references dated to 2019, reflecting ongoing influential discussions in the literature.

### 3.6 Keyword Analysis

To identify research hotspots, we conducted keyword analyses using both CiteSpace and Bibliometrix. (Fig. **8A**) provides a timeline of keyword clustering, pinpointing six major topics: #0 diabetic nephropathy, #1 metabolic syndrome, #2 gestational diabetes mellitus, #3 type 2 diabetes, #4 Alzheimer’s disease, and #5 fibroblasts. Older terms (2014-2015) remain foundational, while recent increases in Alzheimer’s disease- and fibroblast-related keywords highlight expanding research directions. (Fig. **8B)** presents the 20 keywords with the highest citation bursts, with prenatal exposure (8.63) ranking among the strongest, followed by long noncoding RNA (7.06), developmental origins (6.67), rheumatoid arthritis (6.36), and glucose homeostasis (6.19).

Quadrant analysis in Bibliometrix (Fig. **9**) classifies keywords into four categories: Motor themes (*e.g.*, DNA methylation, gene expression, oxidative stress), Niche themes (*e.g.*, insulin resistance, adipose tissue), Emerging or declining themes (*e.g.*, epigenetics, obesity), and Basic themes (*e.g.*, gene expression, mechanisms), and this taxonomy underscores both established research areas and emerging opportunities for further study.

## 4 DISCUSSION

Diabetes mellitus is a major driver of mortality and disability worldwide, affecting diverse populations [20]. Beyond uncontrolled hyperglycemia, DM leads to both acute and chronic complications, imposing significant strain on healthcare systems [21]. Chronic manifestations, such as diabetic nephropathy and neuropathy, are particularly debilitating [22]. Consequently, stringent glycemic control and preventive measures against complications remain top priorities. As an important factor affecting the occurrence and progression of diabetic complications, epigenetic mechanisms have attracted more and more attention from researchers [23, 24].

Drawing on data from 2014 to 2024, our bibliometric analysis underscores a sustained scholarly focus on epigenetic processes in diabetes and its complications. In terms of the number of publications, significant progress has been made in epigenetic research on diabetes and its complications in the past decade, which has revealed a variety of molecular mechanisms and complex regulatory networks in the occurrence and development of a variety of diseases. With the gradual resolution of key scientific problems in this field, the frequency of major theoretical breakthroughs has gradually decreased, and the research paradigm has become mature, which has caused a decline in the number of papers published to some extent. In this context, researchers may explore more cutting-edge and cross-cutting research directions to promote the expansion of disciplinary boundaries and the achievement of innovative breakthroughs. In addition, in the statistical results for the number of articles, articles published in 2024 showed a significant downward trend compared with previous years. However, it should be noted that the data acquisition time node for this study is October 2024, when the annual publication cycle has not yet ended. Due to the lag of academic publication, such as the prolonged review process, online priority publication has not been included in the database, and other factors, the current statistical results may not fully reflect the actual publication situation in 2024. These factors may be important reasons for the low observed data.

In the statistical results, we also found that the H-index and citation frequency showed a continuous downward trend since 2020. Regarding the reasons for the changes in these indicators, the ongoing impact of COVID-19 is indeed a factor that cannot be ignored. In 2021-22, many laboratories around the world faced problems, such as interrupted data collection, blocked international cooperation, and project schedule delays, which directly affected the academic output in the upcoming years. Due to the limited experimental conditions, many researchers have had to adjust their research direction and turn to shorter periods and smaller study subjects. Although some teams still managed to maintain a certain number of publications, the depth and innovation of research may be limited, resulting in a decrease in the proportion of high-quality papers. This trend is reflected in the citation data, which shows that the number of publications is high but the citation growth is weak, and subsequently has a negative impact on the H-index and other indicators measuring academic influence.

In terms of the H-index, the H-index was calculated based on the number of papers published by the researcher and their citation frequency. However, there is a certain lag in citation frequency, leading to a relatively slow update of the H-index. Therefore, the H-index may not reflect the academic impact of research in a timely and accurate manner. Based on the above analysis, although citation frequencies and H-indices declined post-2022, this decrease likely reflects the inherent delay in citation analyses rather than a decline in research quality [25]. From a global perspective, the United States and China have contributed the most publications, with other Western nations cooperating closely [26].

Journal-based metrics indicate that periodicals in endocrinology, metabolism, genetics, and biochemistry dominate this space, led by the *International Journal of Molecular Sciences* [27-29]. Notably, *Diabetologia* stands out for its exceptional influence, as evidenced by its consistently high IF and SJR index, which surpasses many discipline-specific competitors. Indeed, *Diabetologia* emphasizes the core scientific, clinical, and public health dimensions of diabetes, supporting the translation of knowledge into care strategies for patients worldwide [30-32]. This distinction highlights the dual role of *Diabetologia*: it is a catalyst for basic research and a platform for translating discoveries into clinical practice.

Key authors, such as Charlotte Ling and Tina Ronn, have profoundly shaped the field of diabetes epigenetics, elucidating how environmental and metabolic stressors dynamically remodel the epigenome. Their extensive collaborations had not only identified tissue-specific methylation signatures in diabetes but also revealed their reversibility, positioning epigenetic modifications as actionable therapeutic targets [33-37]. Broad collaboration networks, particularly among researchers with more publications, highlight how interdisciplinary approaches accelerate biomarker discovery [38, 39]. Such collaborations will be essential to translate mechanistic insights into therapeutics. We encourage continued cross-institutional and cross-disciplinary efforts to drive innovative research and enhance its translational breadth.

Diabetic nephropathy, as one of the diabetic complications with poor prognosis [40], is a key area of concern in diabetes research due to its high incidence and lethality. The results of the co-citation analysis further confirm the high attention paid by the medical community to chronic complications, especially diabetic nephropathy, which not only reflects the research focus in this field but also reflects the spirit of humanistic care in medical research. Among the top 10 co-cited papers, morphological and mechanistic studies of DNA methylation and related processes have guided contemporary investigations, demonstrating their foundational impact.

Our bibliometric analysis reveals a concentration of research on DNA methylation in diabetic complications, but it is critical to contextualize this within the broader pathophysiological landscape. DNA methylation represents just one facet of a complex interplay between epigenetic mechanisms and inflammatory signaling in diabetes. Recent evidence suggests bidirectional relationships where hyperglycemia-induced oxidative stress triggers aberrant DNA methylation patterns in the promoters of inflammatory genes, while these epigenetic alterations simultaneously modulate ROS production through transcriptional regulation of NADPH oxidase components [41, 42]. This epigenetic-inflammatory circuit extends to proinflammatory cytokine networks, where DNA methylation status of TNF-α, IL-1β, and IL-6 promoters demonstrates tissue-specific alterations in diabetic complications [43]. Particularly compelling is emerging research on epigenetic regulation of inflammasome activation, where hypomethylation of NLRP3 has been observed in diabetic vascular tissues, potentially explaining sustained inflammatory responses despite glycemic control—the “metabolic memory” phenomenon [44]. Importantly, these methylation patterns interact with other epigenetic mechanisms, including histone modifications and non-coding RNAs, to orchestrate inflammatory homeostasis in diabetes. Future research directions should expand beyond DNA methylation to investigate these integrative epigenetic-inflammatory networks, potentially revealing novel therapeutic targets that address the root dysregulation rather than isolated pathways.

Keyword analysis reveals enduring interests in type 2 diabetes, gestational diabetes, and diabetic nephropathy. The focus on epigenetics in the field of diabetes highlights our efforts to reveal how lifestyle and environmental factors interact with genetic susceptibility to diabetes [45]. It is worth noting that epigenetic work related to fibroblasts is emerging as a frontier, transitioning to diabetic complications and tissue fibrosis. Recent studies have pointed out that the dynamic changes in DNA methylation and the characteristics of fibroblasts during the progression of renal fibrosis provide new potential targets for anti-fibrosis therapy [46]. These results are in line with the research direction of tissue remodeling, indicating that by modulating DNA methylation and regulating the function of fibroblasts, it is expected to develop effective anti-fibrosis treatments. Prenatal exposure—a keyword with high burst strength—underscores the importance of maternal factors in determining metabolic outcomes for both mothers and offspring [47]. Maternal hyperglycemia can induce epigenetic memory in fetal tissues, resulting in metabolic dysfunction that persists in the offspring [48]. These findings highlight the need for early-life interventions, such as regulating maternal blood glucose levels or providing nutrients that modulate epigenetics, in breaking the cycle of transgenerational risk.

The thematic landscape of epigenetic research in diabetes reveals distinct developmental clusters, each representing unique mechanistic pathways and translational opportunities. Highly established topics, such as DNA methylation and gene expression, have been shown to have clinical relevance with insulin resistance and β-cell dysfunction [49]. Emerging or Declining Themes, such as epigenetics and obesity, require deeper investigation [50]. This nuanced mapping highlights both entrenched research trajectories and potential avenues for pioneering work.

Our bibliometric analysis not only maps the current landscape of epigenetic research in diabetes but also illuminates critical paths for future investigation and therapeutic development. First, our findings identify several understudied research domains with high potential impact: epigenetic regulation of pancreatic β-cell regeneration, tissue-specific epigenetic signatures as predictive biomarkers for complications, and reversibility of epigenetic modifications through targeted interventions. These areas represent promising frontiers for focused research investment. Second, the international collaboration patterns revealed in our analysis suggest strategic research frameworks that could accelerate discovery, specifically, strengthening connections between clinical centers with large patient cohorts and institutions with advanced epigenomic analytical capabilities would address current methodological limitations. Third, our keyword evolution analysis points toward emerging opportunities for precision medicine approaches in diabetes management. The convergence of epigenetic profiling with metabolomic and proteomic data could enable personalized risk stratification for diabetic complications and tailored therapeutic selection. Notably, our data reveal that histone deacetylase inhibitors and DNA methyltransferase modulators remain understudied in diabetes despite their therapeutic potential demonstrated in other conditions. Future translational research should prioritize evaluating these epigenetic-modifying agents in preclinical diabetic models, particularly focusing on their ability to reverse established complications, and address the crucial clinical challenge of treating patients with longstanding disease.

## 5 STUDY LIMITATION

While our study provides a comprehensive bibliometric analysis of epigenetic research in diabetes, several methodological limitations warrant consideration. The exclusive use of the Web of Science Core Collection database, while ensuring standardized citation metrics, may overlook relevant studies indexed in other major databases such as PubMed or Scopus. This database selection could potentially affect the comprehensive representation of the research landscape, particularly for clinical studies that are more commonly indexed in PubMed. Additionally, our restriction to articles and reviews, although standard for bibliometric analyses, excludes other valuable scholarly outputs, including book chapters and conference proceedings, which may contain early research findings. The English-language limitation, while facilitating consistency in analysis, may inadvertently marginalize significant non-English contributions, particularly from regions with active diabetes research programs. Importantly, while these methodological choices follow established bibliometric practices and enhance analytical consistency, they may introduce some selection bias. However, the robust sample size and the clear emergence of distinct research patterns suggest these limitations do not substantially compromise the validity of our principal findings regarding research trends, geographic distributions, and thematic evolution in diabetes epigenetics. The consistency of our results with known developments in the field further supports the reliability of our conclusions despite these methodological constraints.

## CONCLUSION

This study establishes a bibliometric framework to analyze global trends in diabetes epigenetics research from 2014 to 2024. The analysis reveals distinct geographical leadership, with the United States (31.98% of publications, H-index = 69) and China (19.50%, H-index = 44) emerging as dominant contributors. Three key research clusters —type 2 diabetes, gestational diabetes, and diabetic nephropathy—dominate the field, with studies on DNA methylation and histone modification showing particular therapeutic promise.

Notably, two emerging thematic axes demonstrate strong potential: the connection between diabetes and Alzheimer's disease (r = 0.85, *p* < 0.01) and fibroblast epigenetic reprogramming in tissue repair. These findings suggest a paradigm shift toward exploring neurodegeneration links and stromal microenvironment targeting in diabetic complications.

Our systematic mapping of scholarly activity (n = 1,451 publications), through a multi-level analysis of country contributions, institutional networks, co-cited literature, and keyword evolution, provides an evidence base for strategic research planning.

This structured roadmap identifies both established investigational priorities and emerging opportunities, particularly the clinical translation of locus-specific epigenetic editing technologies, longitudinal Epigenome-Wide Association Studies (EWAS) that capture developmental trajectories, and the integration of multi-omics in diabetic wound healing models. Such directions address current mechanistic knowledge gaps and align with precision medicine frameworks emerging in metabolic disease research. Decoding the epigenetic signature landscape enables targeted investigation, which in turn accelerates the discovery of diagnostic biomarkers and the development of epigenetic therapies.

## AUTHORS’ CONTRIBUTIONS

The authors confirm their contributions to the paper as follows: X.N.: Conceptualization; Y.T., H.Z., L.L., X.G., Q.Z., Y.D., J.Y., R.G., and J.R.: Data curation. Y.T., H.Z., L.L., X.G., Q.Z., Y.D., J.Y., R.G. and J.R.: Formal analysis; Y.T. and X.N.: Funding acquisition; Y.T. Visualization; Y.T.: Writing - original draft; H.Z. and X.N.: Writing - review and editing. All authors reviewed the results and approved the final version of the manuscript.

## Figures and Tables

**Fig. (1):**
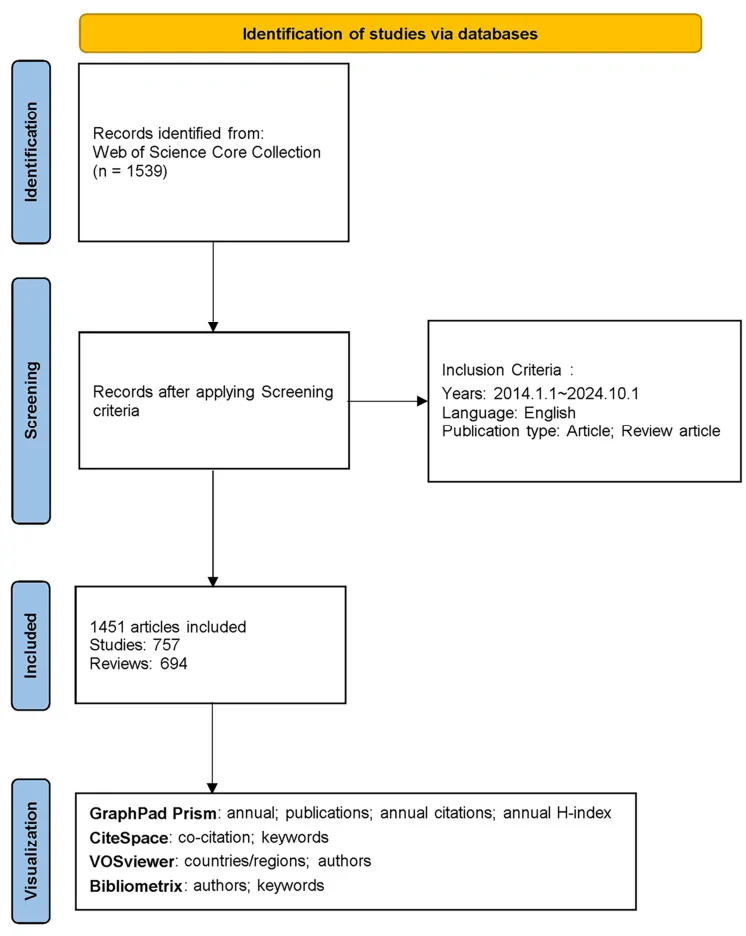
Literature search and data analysis process. Flowchart outlining the systematic literature search strategy implemented to identify relevant publications on epigenetic research in diabetes mellitus from January 1, 2014, to October 1, 2024. Overview of the analytical tools employed during the data analysis phase, detailing the processes for data extraction, visualization, and co-citation analysis.

**Fig. (2):**
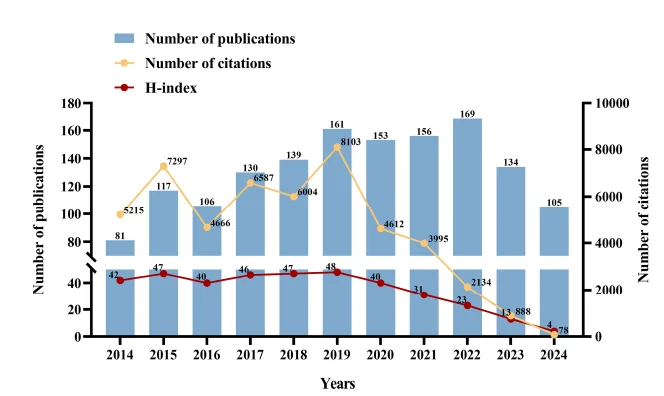
Annual publications, citations, and H-index trends. Graph depicting the annual number of publications, total citations, and H-index trends in epigenetics related to diabetes from 2014 to 2024. This figure highlights the growth of scholarly interest over the years, with a particular focus on the peaks and trends in citation metrics.

**Fig. (3):**
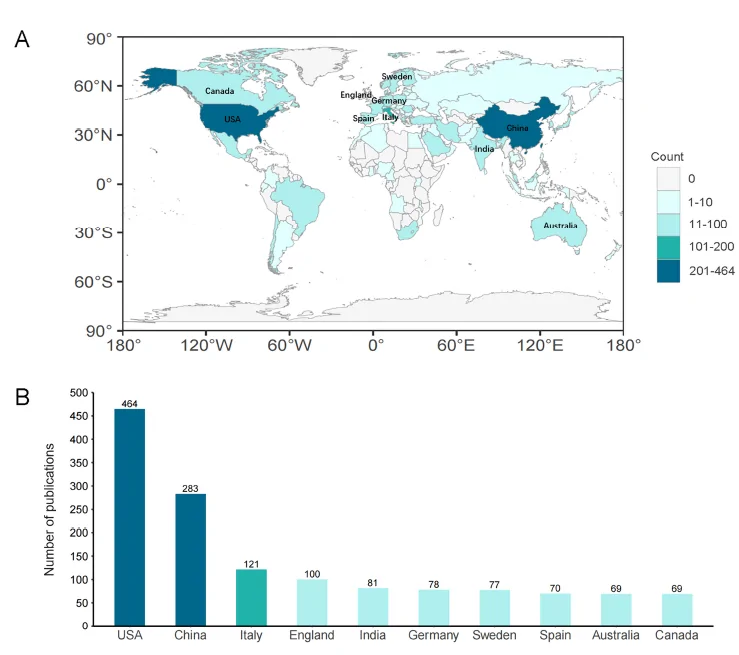
Geographic distribution of research contributions. (**A**) Heatmap showing the geographic distribution of countries involved in research on epigenetics in diabetes mellitus. The size of the circles reflects the number of publications each country has contributed. (**B**) Bar chart displaying the number of publications for the top ten contributing countries, revealing the leading countries in this research area.

**Fig. (4):**
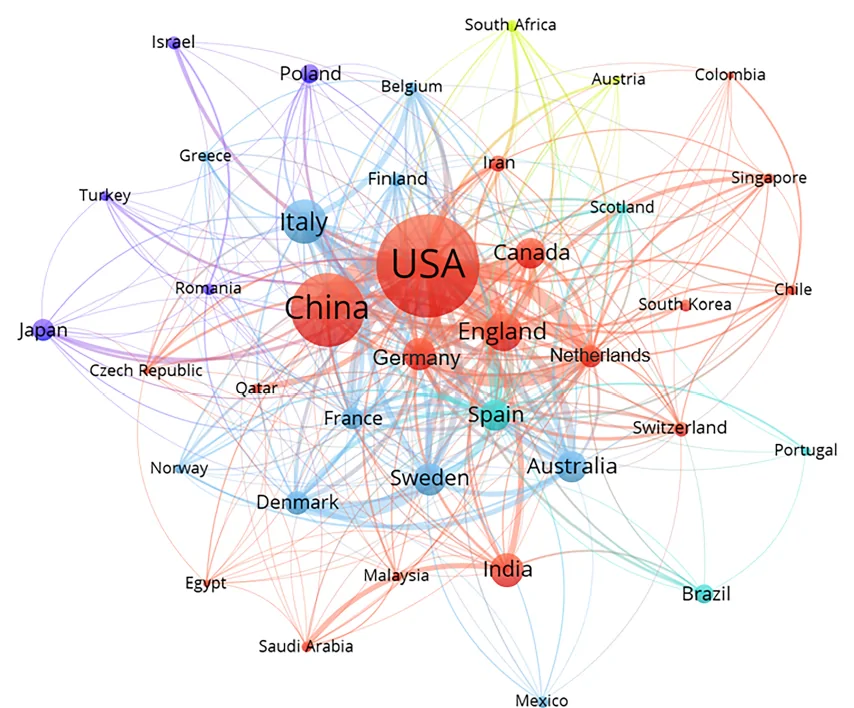
Collaborative network among countries/regions. Network visualization illustrating the collaborative relationships among countries engaged in epigenetic research in diabetes. The size of nodes indicates the volume of publications, while the thickness of edges represents the frequency of collaborations between countries.

**Fig. (5):**
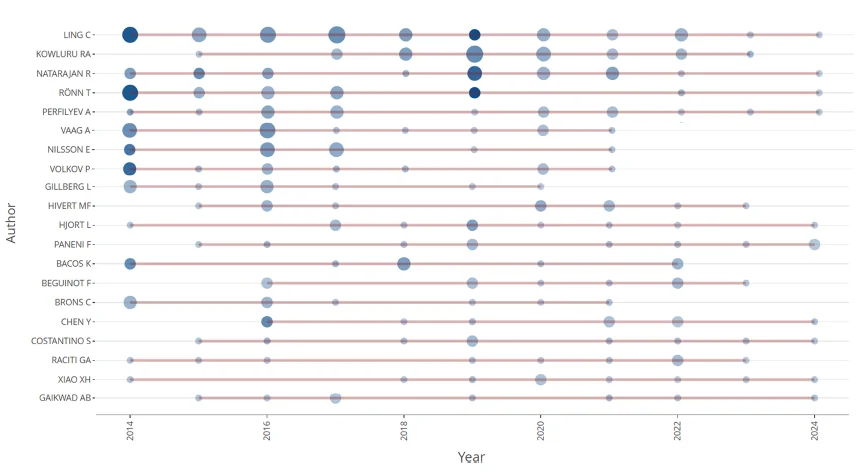
Publication timelines of top authors. Timeline showing the annual publication rates of the top 20 most productive authors in the field of epigenetic research related to diabetes. The pink bars represent each author's output per year, with the size of the bubbles indicating the total number of documents, and bubble color intensity reflecting total citations.

**Fig. (6):**
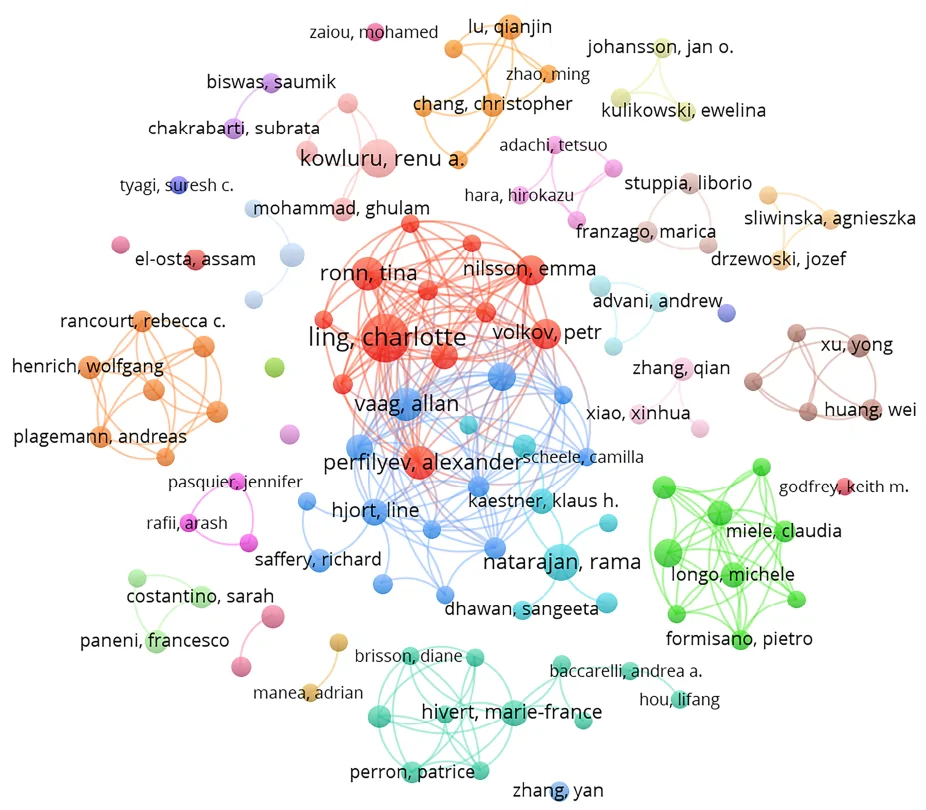
Author collaboration network. Network diagram presenting the cooperation patterns among authors in epigenetics research related to diabetes mellitus. The size of the nodes corresponds to the number of publications by each author, with connecting lines illustrating collaborative links between authors.

**Fig. (7):**
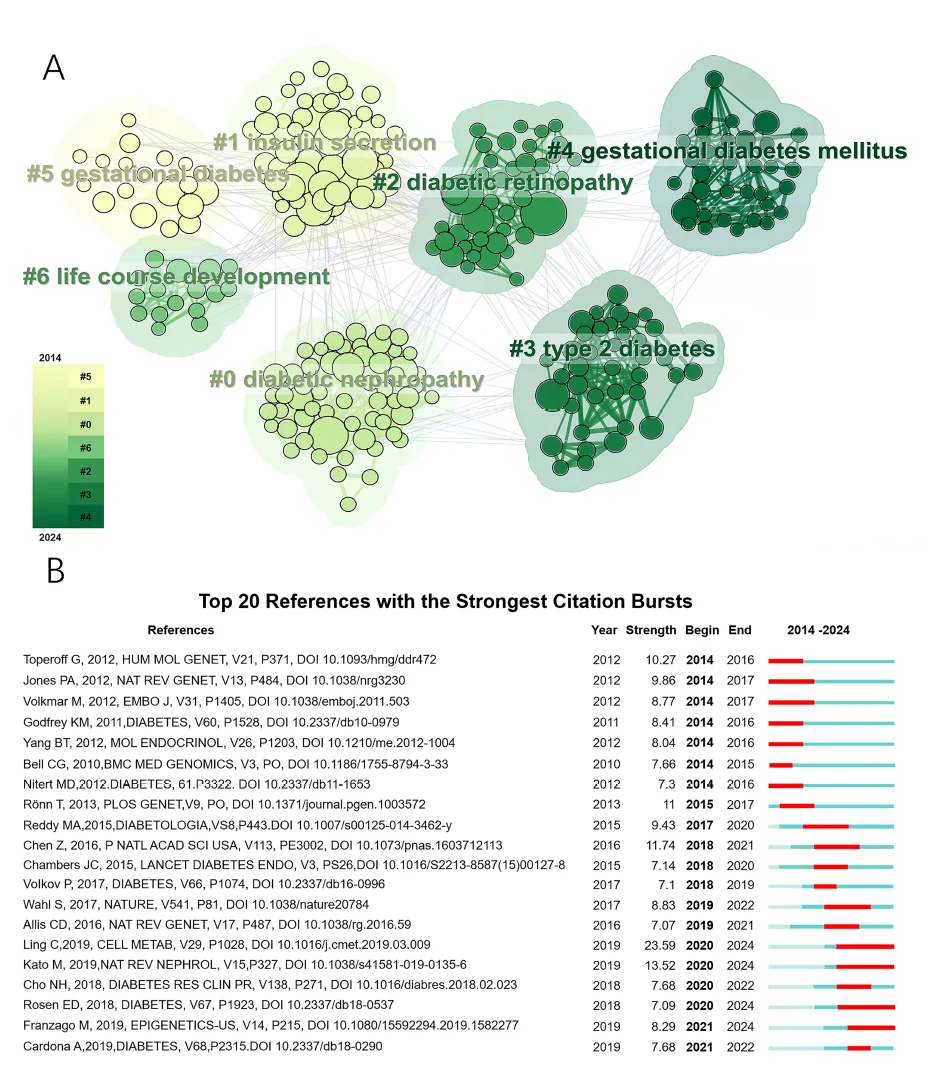
Co-citation analysis of key references. (**A**) Co-citation mapping displaying distinct knowledge clusters among key references in epigenetic studies related to diabetes. The node sizes represent the number of citations each reference has received, while colors correspond to different thematic clusters. (**B**) List of the top 20 references exhibiting significant citation bursts, with Charlotte Ling’s 2019 article showing the highest burst intensity, indicating its major impact on the field.

**Fig. (8):**
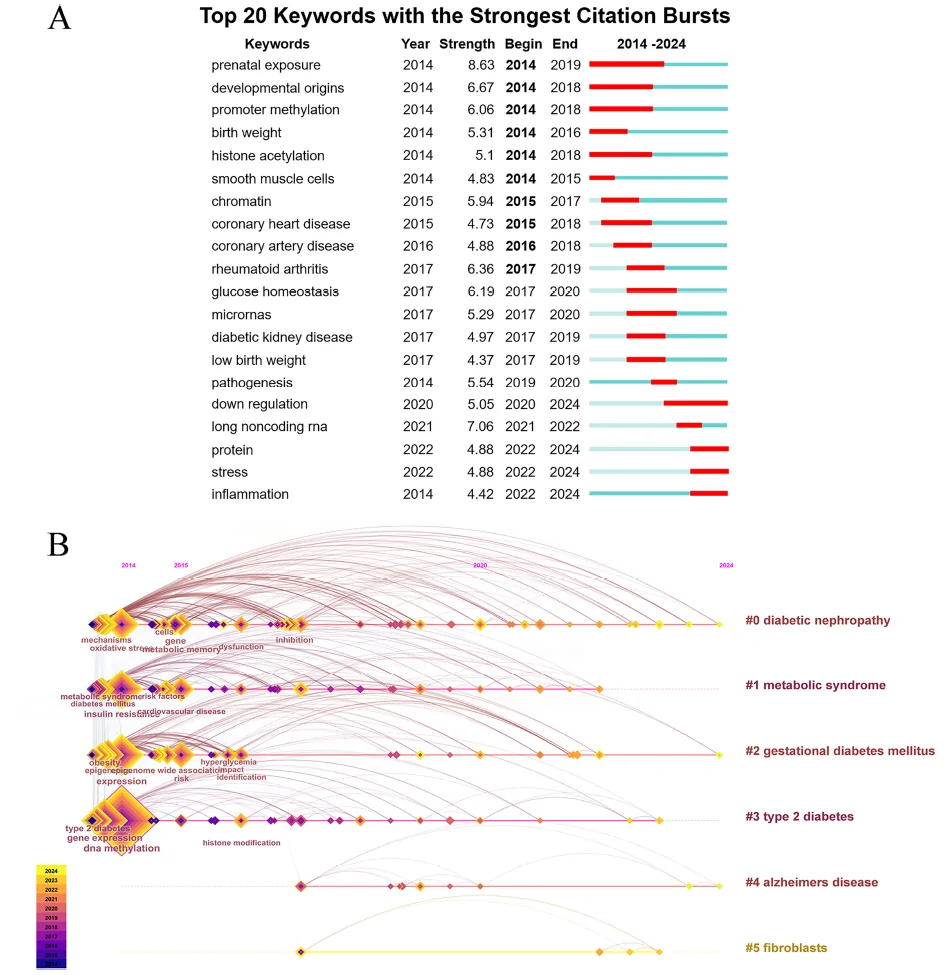
Keyword co-occurrence and trend analysis. (**A**) Timeline graph showing the evolution of major keyword clusters in epigenetic research related to diabetes mellitus across the years. Key themes include diabetic nephropathy, metabolic syndrome, and Alzheimer’s disease. (**B**) Bar chart illustrating the 20 keywords with the strongest citation bursts, emphasizing rising research trends and interests, such as prenatal exposure and long noncoding RNA.

**Fig. (9):**
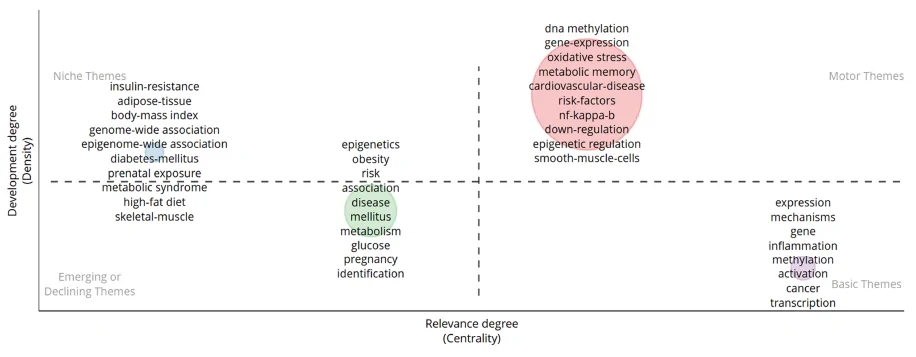
Thematic map of keywords. Thematic map categorizing keywords associated with epigenetics in diabetes into four quadrants: Motor themes (*e.g.*, DNA methylation, insulin resistance), Niche themes (*e.g.*, adipose tissue, metabolic memory), Emerging/declining themes (*e.g.*, obesity, epigenetics), and Basic themes (*e.g.*, mechanisms of gene expression). This mapping provides insight into both well-established and emerging areas of research.

**Table 1:** Top 10 countries/regions by publications, H-index, and citations.

**Rank**	**Country/Region**	**Publications**	**(% of 1451)**	**H-index**	**Total citations**	**Average Citations**
1	USA	464	31.98%	69	19031	41.02
2	China	283	19.50%	44	8012	28.31
3	Italy	121	8.34%	36	3989	32.97
4	England	100	6.89%	36	5326	53.26
5	India	81	5.58%	23	1780	21.98
6	Germany	78	5.38%	34	3145	40.32
7	Sweden	77	5.31%	38	4246	55.14
8	Spain	70	4.82%	28	2529	36.13
9	Australia	69	4.76%	31	3498	50.7
10	Canada	69	4.76%	29	2584	37.45

**Table 2:** Top 10 journals with the most publications.

**Rank**	**Journal**	**Publications**	**IF_2023_**	**SJR Indicator_2023_**	**JCR**
1	International Journal of Molecular Sciences	66 Article: 13 Review article: 53	4.9	1.179	Q1: Biochemistry and Molecular Biology Q2: Chemistry, Multidisciplinary
2	Clinical Epigenetics	51 Article: 42 Review article: 9	4.8	1.727	Q1: Genetics and Heredity, Oncology
3	Frontiers in Endocrinology	48 Article: 20 Review article: 28	3.9	1.240	Q2: Endocrinology and Metabolism
4	Diabetologia	32 Article: 20 Review article: 12	8.4	3.355	Q1: Endocrinology and Metabolism
5	Nutrients	26 Article: 7 Review article: 19	4.8	1.301	Q1: Nutrition and Dietetics
6	Frontiers in Genetics	23 Article: 12 Review article: 11	2.8	0.853	Q2: Genetics and Heredity
7	Epigenetics	19 Article: 16 Review article: 3	2.9	1.149	Q2: Genetics and Heredity Q3: Biochemistry and Molecular Biology
8	Epigenomics	19 Article: 13 Review article: 6	3.0	0.751	Q2: Genetics and Heredity
9	Current Diabetes Reports	17 Article: 1 Review article: 16	5.2	1.758	Q1: Endocrinology and Metabolism
10	Frontiers in Immunology	17 Article: 4 Review article: 13	5.7	1.868	Q1: Immunology

**Table 3:** The top 10 influential authors.

**Author**	**Publications**	**Citations**	**Average Citations**	**H-index**
Ling, Charlotte	30	2549	84.97	25
Kowluru, Renu A.	21	747	35.57	17
Natarajan, Rama	19	1705	89.74	13
Ronn, Tina	17	1919	112.88	15
Perfilyev, Alexander	16	815	50.94	10
Vaag, Allan	15	959	63.93	14
Nilsson, Emma	12	1127	93.92	11
Volkov, Petr	11	1190	108.18	10
Gillberg, Linn	10	402	40.20	9
Hjort, Line	10	369	36.90	7

**Table 4:** Top 10 highly co-citation references.

**Rank**	**Title**	**Count**	**Year**	**Document Type**	**Source**
1	Epigenetics in human obesity and type 2 diabetes	65	2019	Review	Cell metabolism
2	Epigenome-wide association study of body mass index and the adverse outcomes of adiposity	51	2017	Article	Nature
3	Epigenomic profiling reveals an association between persistence of DNA methylation and metabolic memory in the DCCT/EDIC type 1 diabetes cohort	47	2016	Article	Proceedings of The National Academy of Sciences of The United States of America
4	Functions of DNA methylation: Islands, start sites, gene bodies, and beyond	37	2019	Review	Nature Reviews Genetics
5	Epigenetics and epigenomics in diabetic kidney disease and metabolic memory	34	2019	Review	Nature Reviews Nephrology
6	DNA methylation profiling identifies epigenetic dysregulation in pancreatic islets from type 2 diabetic patients	33	2012	Article	Embo Journal
7	Epigenetic mechanisms in diabetic complications and metabolic memory	30	2014	Review	Diabetologia
8	A six-month exercise intervention influences the genome-wide DNA methylation pattern in human adipose tissue	30	2013	Article	Plos Genetics
9	Genome-wide survey reveals predisposing diabetes type 2-related DNA methylation variations in human peripheral blood	28	2012	Article	Human Molecular Genetics
10	Epigenome-wide association of DNA methylation markers in peripheral blood from Indian Asians and Europeans with incident type 2 diabetes: a nested case-control study	24	2015	Article	Lancet Diabetes & Endocrinology

## LIST OF ABBREVIATIONS

DM: Diabetes Mellitus
IDF: International Diabetes Federation
EWAS: Epigenome-Wide Association Studies

## STANDARDS OF REPORTING

PRISMA-ScR guidelines were followed.

## SUPPLEMENTARY MATERIAL

PRISMA-ScR checklist is available as supplementary material on the publisher’s website along with the published article.
